# Supplementary material for: Asymmetric Synthesis of Quaternary Hydantoins via a Palladium-Catalyzed Aza-Heck Cyclization
Source: J Am Chem Soc. 2025 Nov 14;147(49):44692–8. doi: 10.1021/jacs.5c16022 (PMC12703750; doi:10.1021/jacs.5c16022)
Supplement: Supplementary file 2 [file ja5c16022_si_002.zip › All NMR FID Files/S8 2D Spectra/S8 Conformer 2 COSY/4/pdata/1/email_EM06-035-isomer#2-1COSY_4_1.pdf]

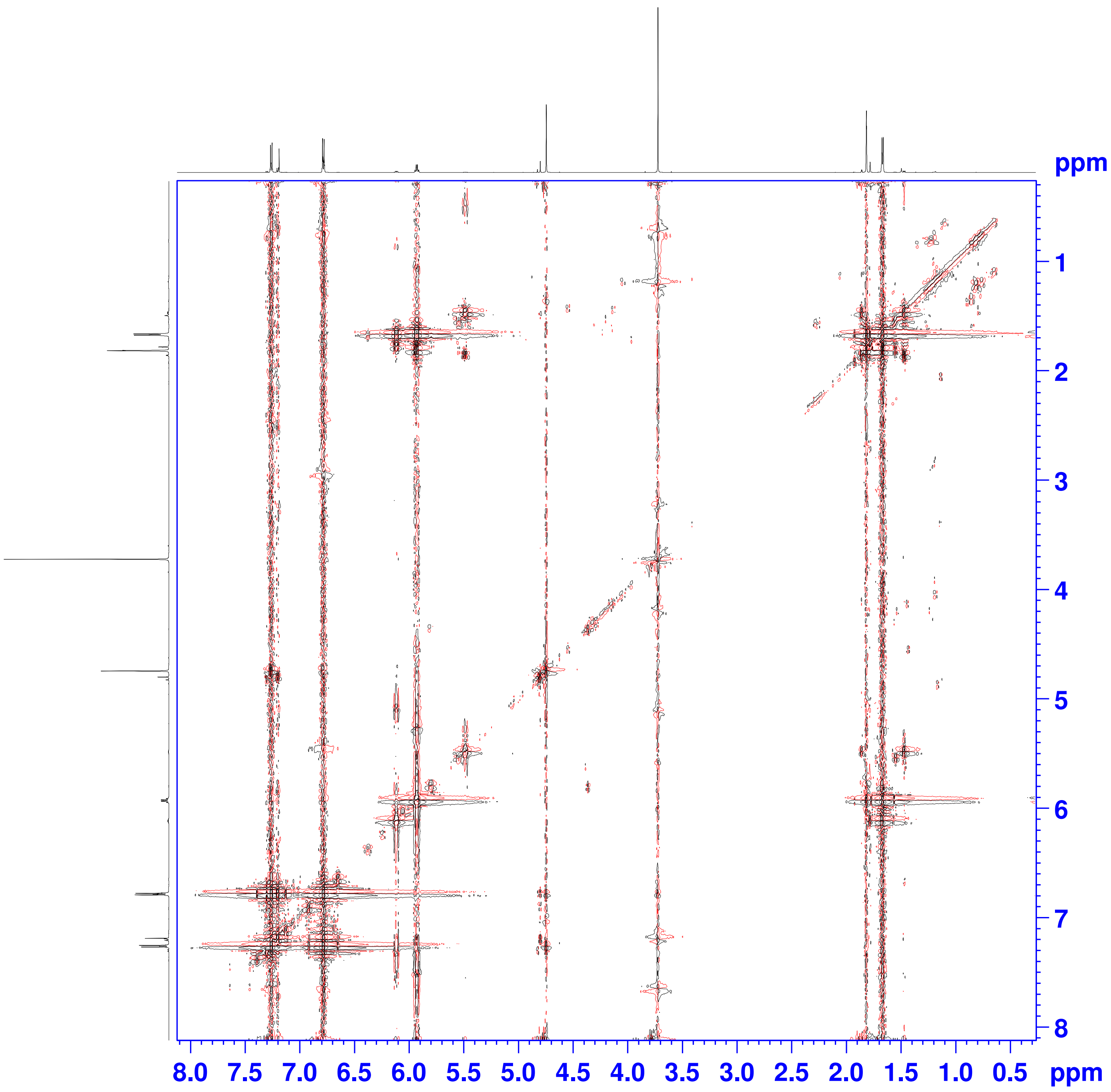

BRUKER

```
Current Data Parameters
NAME       EM06-035-isomer#2-1COSY
EXPNO                     4
PROCNO                    1

F2 - Acquisition Parameters
Date_          20250211
Time              15.13 h
INSTRUM  Avance Neo 600 at University of Delaware
PROBHD   Z185170_0001 (
PULPROG    cosygpmfphpp
TD                 2048
SOLVENT            CDCl3
NS                    4
DS                   16
SWH           4716.981 Hz
FIDRES        4.606427 Hz
AQ           0.2170880 sec
RG                  101
DW              106.000 usec
DE                10.00 usec
TE                298.0 K
D0           0.00009572 sec
D1           1.91193604 sec
D11          0.03000000 sec
D12          0.00002000 sec
D16          0.00020000 sec
IN0          0.00021200 sec
TDav                  1
SFO1        600.1325742 MHz
NUC1                 1H
P1                 8.07 usec
P2                16.14 usec
P17             2500.00 usec
PLW1         6.90000010 W
PLW10        0.71898001 W
GPNAM[1]     SMSQ10.100
GPZ1              10.00 %
GPNAM[2]     SMSQ10.100
GPZ2              20.00 %
P16             1000.00 usec

======== F1 INDIRECT DIMENSION ========
td1       256
sw_F1     7.859899

F1 - Acquisition parameters
TD                  256
SFO1           600.1326 MHz
FIDRES        36.851414 Hz
SW                7.860 ppm
FnMODE      States-TPPI

F2 - Processing parameters
SI                 1024
SF          600.1300578 MHz
WDW               QSINE
SSB                   2
LB                    0 Hz
GB                    0
PC                 1.40

F1 - Processing parameters
SI                 1024
MC2         States-TPPI
SF          600.1300578 MHz
WDW               QSINE
SSB                   2
LB                    0 Hz
GB                    0
```
